# Hydrological drought forecasting and monitoring system development using artificial neural network (ANN) in Ethiopia

**DOI:** 10.1016/j.heliyon.2023.e13287

**Published:** 2023-01-29

**Authors:** Kassa Abera Tareke, Admasu Gebeyehu Awoke

**Affiliations:** a Wollo University, Kombolcha Institute of Technology, KioT, Ethiopia; b Addis Ababa University, Addis Ababa Institute of Technology, AAiT, Ethiopia

**Keywords:** Artificial neural network, Hydrological drought forecasting, Linear scaling, Regional climate model

## Abstract

The objective of this study is to investigate and perform long–term forecasting of both streamflow and hydrological drought over Ethiopia. Observed streamflow and precipitation data are collected from 17 streamflow stations and 34 rainfall gauge stations to forecast future streamflow and hydrological drought from 2026 to 2099. Streamflow forecasting is performed using an artificial neural network (ANN) in conjunction with python software. Observed precipitation and streamflow data from 1973 to 2014 are used to train and test the ANN model by 70 and 30% ratios, respectively. After training the model, future downscaled precipitation data from regional climate models (RCM) have been used as input data to forecast future streamflow. Three RCM models were used to downscale historical and future climate data. RACMO is found a good downscaling model for all selected stations. The linear scaling bias correction technique results in less than 2% error compared to other alternative techniques. The result indicates that ANN is a good tool to forecast streamflow in areas having a good correlation between precipitation and streamflow such as Abbay, Awash, Baro, Omo Gibe, and Tekeze river basins. But in arid areas for example Genale Dawa, Wabishebele, and Rift Valley basins, the model is not suitable because the input data (precipitation) have high variation than the output variable (streamflow). In such areas, meteorological drought analysis and forecasting are better than hydrological drought analysis. Finally, future hydrological drought is analyzed using forecasted streamflow data as input to the streamflow drought index (SDI). The result indicates that 2028, 2036, 2042, 2044, 2062, and 2063 are the expected extreme drought years in most river basins of Ethiopia in the future. This shows that at least one extreme drought is expected in each decade in the future. Therefore, extensive research in drought analysis and forecasting is needed to develop an effective drought early warning system, and water resource management policy.

## 1 Introduction

Drought results from a prolonged period of precipitation deficiency and shortage of surface and subsurface water availability [[1], [2], [3], [4]]. The definition of drought still depended on different perceptions but structurally it is categorized as meteorological, agricultural, hydrological, and socio-economic drought based on deficiency of precipitation, lack of surface and sub-surface water availability, and unfair water distribution between supply and demand [5].

Drought monitoring and forecasting play a great role in water resource management and early warning development system for drought and flood hazard mitigation. Artificial Neural Networks are now widely applied in a broad range of fields, including image processing, signal processing, medical studies, financial predictions, power systems, and pattern recognition among others [6]. Recently, several civil engineering problems such as structural damage analysis [7], material strength prediction [8], buckling, and energy trapping in building blocks [9] are solved using artificial neural networks. At the same time, ANN is also used in many aspects of hydrological and meteorological studies such as streamflow forecasting, groundwater analysis, precipitation forecasting, rainfall-runoff modeling, and water quality issues, flood and drought forecasting [10]. Development in forecasting and early warning of drought phenomena is increasingly applied in many regions of the world. This is being done to mitigate the consequences of drought in vulnerable river basins and to save human life [11].

Different drought modeling and forecasting techniques are in use today. The seasonal autoregressive integrated moving average model (SARIMA), the Adaptive Neuro-Fuzzy inference system, the Markov chain model, the Log-Linear model, and the Artificial Neural Network (ANN) model are some of the common drought-predicting models [[12], [13], [14], [15], [16]]. Historically, time series-based statistical models for hydrologic drought forecasting have been applied. Regression models and autoregressive moving average (ARMA) models are typical models for statistical time series methods for forecasting. They are linear models, nevertheless, and they can only partially capture non-stationaries and non-linearity in the hydrologic data since they assume that the data are stationary [17]. But the traditional statistical time series analysis in hydrology has several limitations related to non-linear variables. This problem is now overcome and improved by using robust time series predictive techniques like ANN [18]. Imprecise in nature, uncertainty, lack of data, and inconsistency, the physical characteristics of the region have a great influence on meteorological and hydrological variables in Ethiopia. In such circumstances, Fussy Logic techniques are renowned to be highly enhancing the modeling of such natural dynamics and variability [[15], [16], [17]]. Drought is the most destructive natural phenomenon resulting from climate change it is usually important to analyze and monitor it on a regional scale. The fuzzy logic predictive system among the various available Artificial Intelligence techniques emerges as an advantageous technique in forecasting future hydroclimatic events such as floods and droughts [[19], [20], [21], [22], [23], [24]]. However, due to their inherent nonlinear nature and modeling flexibility, artificial neural networks (ANNs) have recently demonstrated tremendous capacity in modeling and forecasting nonlinear and non-stationary time series in hydrology and water resource engineering [17]. Among different drought forecasting models, the ANN model is appropriate for large and complex data sets to analyze and forecast [25].

The majority of water resource variables reveal a highly nonlinear behavior because of spatial and temporal variations [26]. Therefore, to solve these nonlinear variables, one of the most attractive features is ANN modeling which can learn the exact behavior between the inputs and outputs from the examples without any kind of physical involvement [27]. Many applications of ANNs for prediction, forecasting, modeling, and estimation of water resource variables (i.e. water discharge, sediment discharge, rainfall-runoff, groundwater flow, precipitation, forecasting, water quality, etc.) have been found and related to river discharge and sediment [28]. In recent years, ANNs have been used intensively for prediction and forecasting in several water-related areas, including water resource study and drought forecasting [29]. It is clear that artificial neural networks constitute an emerging new technology, and their full potential for solving hydrologic problems must be explored further. ANN received a great deal in several hydroclimatic events in floods and drought forecasting for appropriate early warning system development [[30], [31], [32], [33], [34]].

Every year drought has occurred in at least one or more countries in the world. This hydrological event has been widely concerned on water quality and water scarcity. Because hydrological drought can export or import dissolved organic carbon (DOC). However, the response relationship of DOC to hydrological drought characteristics interms of duration and severity requires in-depth research [35]. Many drought forecasting investigations indicated that artificial neural network is widely used in most water-related research due to their superior ability to forecast hydrological events from non-linear variables [36]. There are many drought indices used to forecast future drought variability like standardized precipitation index (SPI), palmer drought severity index (PDSI), normalized vegetation index (NDV), and standardized streamflow index (SDI). The former three indices are used for meteorological and agricultural drought analysis while the latter fourth index is used for hydrological drought analysis. Recently using remote sensing instruments NVI is used to forecast drought severity but it has a limitation in areas that have no good vegetation coverage [19]. Since the objective of this study is focused on hydrological drought forecasting related to climate change and SDI is selected for analysis. However, the future SDI value is directly dependent on future streamflow variation and it is important primarily to forecast streamflow to estimate SDI values. Therefore, observed precipitation data at various stations near to streamflow station was used as input and a single streamflow station data was used as output to train and test ANN model performance. The statistical performance criteria such as root mean square error (RMSE), coefficient of determination (R^2^), and mean absolute percentage error (MAPE) was used to check the performance of the ANN model prediction.

The major drawback of present studies in drought forecasting is using the same variable to forecast future drought conditions [15,37,38]. Several scholars tried to forecast meteorological and hydrological droughts using ANN by introducing drought indices value as input data for the architecture of ANN [[39], [40], [41]]. Those scholars used the SPI time series value as input for the ANN input layer to predict one month or two months ahead. A statistical error computation result from a similar variable will result in a good performance. But it does not give good information about the nonlinear problems. Therefore, this study is aimed to forecast future hydrological drought by integrating two different variables (precipitation and streamflow data). The historical precipitation data were used as input and historical streamflow as output in the model training. After checking the performance of ANN model streamflow prediction, the future streamflow was forecasted using downscaled precipitation data. Then the hydrological drought condition from 2026 to 2099 is forecasted using future streamflow values.

Surface and sub surface water resources are directly related to streamflow variability. So streamflow forecasting has a great role in reservoir optimization development to mitigate future drought impact on the society [42]. Besides to this streamflow forecasting using ANN is very important in water resource planning and management under poor gauged river basins [43]. In streamflow timeseries analysis both peack flow and low flow are the important issues in hydrology to develop strategical water resource management during hydrological events such as floods and droughts [44]. Therefore, in this study streamflow is forecasted inorder to predict the future drought condition in Ethiopia.

## 2 Materials and method

### 2.1 Description of the study area

**Fig. 1:**
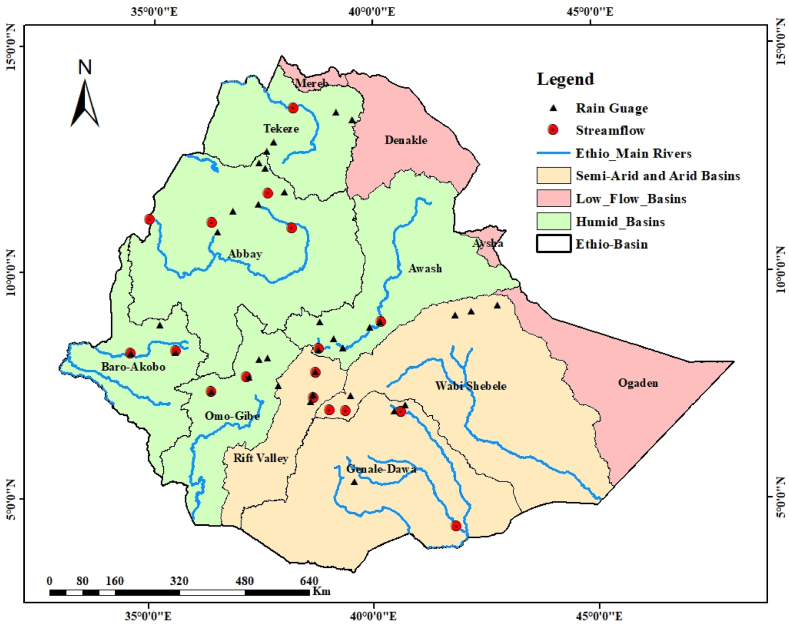
Major River basins in Ethiopia and spatial location of selected hydroclimatic stations.

Ethiopia is geographically located in East Africa at a latitude of 3°–15° N and a longitude of 33° - 48° E [45]. There are twelve major river basins of which four are now categorized under low flow to dry basins (Mereb, Denakil, Ayisha, and Ogaden). Therefore, this study was focused on eight river basins; five are dominated by humid to temperate climate zone (Omo Gibe, Baro Akobo, Abbay, Awash, and Tekeze), respectively whereas the remaining three are dominated by semi-arid to arid climate zone (Genale Dawa, Rift Valley, and Wabishebele), respectively [[46], [47], [48]]. Fig. 1 shows the different climatic zones and the spatial location of selected hydrometeorological stations in the study area.

In the previous few decades, drought studies were conducted in some parts of Ethiopia with a special focus on meteorological drought assessment [49]. However, hydrological drought analysis and long-term drought forecasting using the recent data-driven application are still not addressed. Therefore, this study addressed both streamflow forecasting and hydrological drought forecasting based on the historical observed data trend and future downscaled climatic data.

### 2.2 Data collection and preparation

Ethiopian Meteorology Institute (EMI) and the Ministry of Water and Energy (MoWE) provided daily precipitation and streamflow data, respectively. Observed rainfall data were collected from 42 stations and streamflow was from 32 stations from 1973 to 2014. However, only 34 rainfall stations and 17 streamflow station show good correlation inorder to forecast future streamflow. The monthly rainfall was used as input and the mean monthly streamflow was used as an output for training and testing the artificial neural network (ANN) model using python software. Downscaled precipitation data were also used for future forecasting of streamflow in all river basins from 2026 to 2099. Table 1 gives the geographical location of both hydrological and meteorological stations with some physical characteristics (Area and elevation) and the mean annual flow and annual rainfall of selected stations. Backpropagation multilayer perceptron (BPMLP) was used to predict future streamflow from past rainfall and streamflows.

**Table 1:** Basic statistical data of selected hydroclimatic stations.

Hydrological Stations					
Basin	Stations	Latitude (^o^)	Longitude (^o^)	Area (Km^2^)	Mean Annual flow (m^3^/s)
Abbay	Gilgel Beles	11.17	36.37	675	212.2
	Border	11.23	34.98	17,254	29168.9
	Gummera	11.83	37.63	1394	412.7
	Kessie	11.07	38.18	65,784	6192.4
Awash	Awash7	8.98	40.18	19110.8	696.4
	Hombel	8.38	38.78	7656	507.8
Baro	Gambela	8.25	34.58	23,461	4770.0
	Sorie	8.32	35.60	1622	608.7
Genale Dawa	Halewel	4.43	41.83	54,093	1893.3
	Weib	6.98	40.62	3576.9	135.9
Omo Gibe	Assendabo	7.75	37.18	2966	14696.1
	Gojeb	7.42	36.38	3577	22254.3
Rift Valley	Dedessa	7.28	38.67	156	13.4
	Kekersitu	7.85	38.72	7488	59.6
Tekeze	Embamadre	13.73	38.20	45,694	3084.7
Wabishebele	Wabi@bridge	7.02	39.03	1035	89.0
	Leliso	7.00	39.38	135	21.0
Meteorological Stations					
Basin	Stations	Latitude	Longitude	Elevation (m)	Annual rainfall (mm)
Abbay	Bahir Dar	11.60	37.42	1770	1423.9
	Gondar	12.52	37.43	1973	1161.3
	Debre Tabor	11.87	38.00	2612	1536.0
	Makisegnit	12.39	37.56	1912	1003.9
	Dangila	11.43	36.85	2116	1609.6
	Chagni	10.97	36.50	1614	1718.3
Awash	Addis Ababa	8.98	38.80	2354	1042.8
	Mojo	8.61	39.11	1763	953.8
	Awash7	8.98	40.15	923	617.4
	Metehara	8.86	39.92	944	520.7
	Melkawerer	8.40	39.32	1540	848.2
	Hombel	8.37	38.77	1743	863.8
Baro	Alemteferi	8.90	35.23	1630	1636.4
	Gambela	8.25	34.58	500	1162.6
	Metu	8.28	35.57	1711	1629.3
Genale Dawa	Ginir	7.13	40.70	1750	1128.4
	Goro	7.00	40.47	1800	938.0
	Negele	5.42	39.57	1544	690.0
Omo Gibe	Abelti	8.17	37.63	1968	1068.5
	Wolkite	8.13	37.45	2000	1232.5
	Assendabo	7.75	37.22	1764	1181.2
	Gojeb	7.42	36.38	1250	1500.3
Rift Valley	Arsi	7.35	38.65	1800	845.8
	Adamitulu	7.86	38.70	1653	836.4
	Hosana	7.55	37.87	2200	1162.2
	Shashemene	7.20	38.60	2080	791.5
Tekeze	Dabat	12.98	37.75	2685	947.3
	Hagereselam	13.65	39.17	2618	812.3
	Mekele	13.47	39.53	2257	591.2
	Ambagiorgis	12.77	37.60	2900	976.0
Wabishebele	Bisidimo	9.20	42.20	1669	717.9
	Girawa	9.13	41.83	2100	973.4
	Jijiga	9.33	42.78	1775	588.4
	Endeto	7.34	39.49	2480	887.9

Once the relevant data has collected, missing data were filled using arithmetic mean method and normal ratio method based on the normal annual rainfall percentage deviation with neighbor stations. In the case of Abbay, Awash, Baro, Omo Gibe, and Tekeze river basins, the percentage of missing rainfall data were insignificant. But in the arid river basins such as Genale Dawa, Rift Valley and Wabishebele the missing was significant. After filling some missed data, the consistency and homogeneity test was conducted to all stations using double mass curve and non-dimensional ratio method, respectively and the analysis revealed that all selected stations considered for streamflow forecasting and future drought analysis.

### 2.3 Downscaling climatic data and bias correction

For the last five and six decades, several researchers have conducted climate change investigations in different parts of the world. However, local climate changes force different sights of understanding the effect of natural disasters related to climate change. Since the successful understanding of local climate change impact has a great role in climate adaptation and early warning system development for drought and flood mitigation. Future and past (Historical) daily precipitation data were projected from Global Climate Models (GCM)and Regional Climate Model (RCM) from the African domain using CORDEX projects. Since the GCM data spatially covers a large area and its resolution is coarser [50], so the data were generated from RCM for the Africa domain (AFR-44) historically from 1965 to 2005 and the future was from 2026 to 2100 using the RCP4.5 climate change scenario. MIROC5, KNMI, and SMHI were the driving GCM models, and RCA4, RACMO, and RCA were the corresponding RCM models selected for this study. Both the historical and future climate data were downscaled from Coupled Model Intercomparison Project Phase 5 (CHIP5). The statistical downscaling model (SDSM) was applied to project the historical and future climate data due to its superior ability to capture local scale climate variability [[51], [52], [53]]. After bias correction using Climate Model data for the hydrological modeling (CMhyd) tool, the three models’ data generation performance was checked by the coefficient of determination (R^2^), and mean absolute percentage error (MAPE) using overlap year data from 1973 to 2005. Five bias correction techniques were evaluated in this study. These are delta change correction (DCC), precipitation local intensity scaling (PLIS), linear Scaling (LS), power transformation (PT), and distribution mapping for Precipitation (DMP). Finally, by comparing their percentage of error, an appropriate bias correction technique is selected.

### 2.4 Streamflow forecasting using artificial neural network

Climate change is the primary cause of hydroclimatic events, floods, and drought. This climatical variability can directly affect the natural water availability such as streamflow, groundwater, reservoir, lake, etc. [[54], [55], [56]]. Hydrological drought monitoring and forecasting are associated with surface and subsurface water availability. However, future streamflow forecasting is a challenging task for researchers due to its spatial and temporal variability. Drought affects the natural environment of an area when it persists for a longer period. Therefore, drought forecasting is crucial for river basin planning and management of its water and natural resource systems. In the previous ten years, neural networks have demonstrated excellent modeling and forecasting capabilities for nonlinear and non-stationary time series [57].

Recently there are several physical-based hydrological models to predict streamflow. But most of them are required intensive input data and complex mathematical algorisms. Because the transformation process from rainfall to streamflow is complex and non-linear. Since the rainfall distribution can be influenced by temporal and spatial factors, such as duration of rainfall, and catchment characteristics. Therefore, this complex and non-linear process is not easily described by a simple model. Nowadays, the application of Artificial intelligence and Machine learning is becoming a prominent tool for many aspects of water resource studies [[58], [59], [60], [61]].

**Fig. 2:**
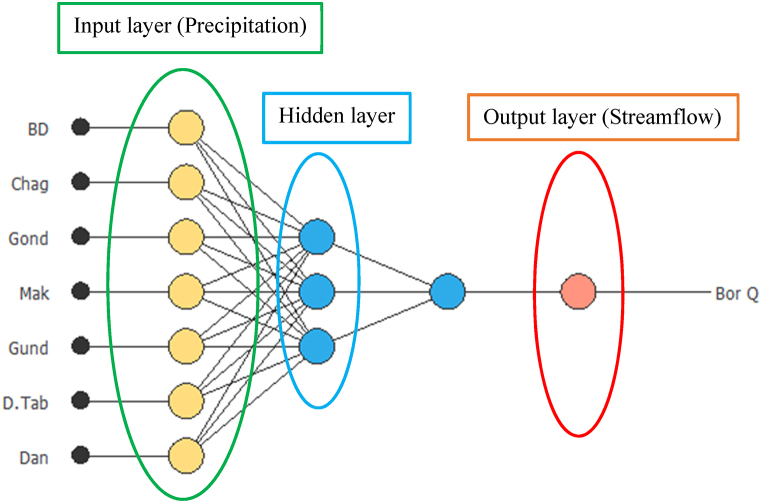
A three-layer feedforward neural networks Architecture for streamflow forecasting.

In this study, ANN was applied to explore simulating the nonlinear hydrologic behavior of eight river basins to forecast future hydrological drought. In the development of ANN arichtecture, input selection and decision on number of hidden laye and perceptron plays a great role in the model output performance [62]. In each river basin, the input data were selected by checking historical observed precipitation and streamflow correlation. Out of 42 rainfall stations and 32 streamflow stations, only 34 rainfall and 17 streamflow stations were show good correlation. Therefore, two ore more rainfall station are used as input to forecast streamflow at a single station. Forty-two years of consecutive historical monthly precipitation and streamflow data were applied to train and test the ANN model. The first thirty years of data (360 months) were used for model training (70%), while the remaining twelve years of data (204 months) were used for the evaluation of model performance (30%). The architecture of the ANN structure contains three layers (input, hidden, and output), respectively (see Fig. 2). To forecast the future condition of drought, first, the model is well-trained, tested, and validated using historical observed data, and the performance of the model was checked by coefficient determination (R^2^), root means square error (RMSE) and mean absolute error (MAE) with the observed data. After the train and test reached recommendable statistical performance, 75-year future precipitation data (2026–2100) was used as an input to forecast future streamflow. Fig. 2 is an illustrative example of one streamflow station forecasting in the Abbay river basin. Seven precipitation stations were used as input and streamflow at the Border station was used as the output variable. In the same manner, the remaining streamflow stations in this study were computed in the same procedure as the Border station.

The performance of forecasting streamflow using ANN was performed by trial and error approach. The important parameters considered in trial and error are epoch, batch size, and the number of perceptrons in the hidden layer. After many trials, epoch and batch size was fixed as 100 and 64, respectively. A small number of neurons in the hidden layer results in underfitting whereas large numbers result in overfitting. Therefore, by trial and error procedure, appropriate numbers of neurons in the hidden layer were selected for each specific station. All the input and output variables were standardized from 0 to 1, to illuminate the time and spatial variability problem. The sigmoid function was applied between the input and hidden layer whereas a linear regression function was applied between the hidden and output layer. The sigmoid function is mainly used as an activation function in various layers of neural networks. The estimation of the sigmoid function in a backpropagation neural network approach is given by Equation (1) [63]. If the value of x is more negative, the output is approximately 0; and if the value of x is more positive, the output is approximately 1.

1 $$\sigma \left(x\right)=\frac{1}{1+e^{-x}}$$

Where σ(x) is sigmoid function

The main difficulty in the artificial neural network is deciding the number of hidden layers and the number of perceptrons in each hidden layer. Increasing the number of hidden layers makes the ANN architecture more complex and the performance of the overall system is reduced. Therefore, a single hidden layer is mostly recommended by scientists [64].

### 2.5 Model performance measures

Statistically, there are several models of performance measuring techniques. Root Mean Square Error (RMSE), Coefficient of determination (R^2^), and Mean Absolute Percentage Error (MAPE) are the most common performance measures. Those three techniques were adopted for this study to check the performance of the backpropagation multi-linear perceptron (BPMLP) model to forecast monthly streamflow using monthly precipitation data as model input. The performance evaluation of each technique is described by Equations 2, 3), AN 4, respectively [[65], [66], [67]].

2 $$RMSE=\sqrt{\sum_{i=1}^{n}\frac{{\left(Q_{obs}-Q_{sim}\right)}^{2}}{N}}$$

3 $$r=\frac{n\left(\sum Q_{obs}*Q_{sim}\right)-\left(Q_{obs}\right)\left(Q_{sim}\right)}{\sqrt{\left[n\sum {Q_{obs}}^{2}-{\left(\sum Q_{obs}\right)}^{2}\right]}\left[n\sum {Q_{sim}}^{2}-\left(\sum {Q_{sim}}^{)}\right)\right]}$$

Where coefficient of determination (R^2^) = coefficient of correlation square (r^2^)

4 $$MAPE=\frac{1}{N}\sum_{i=1}^{n}\left|\frac{Q_{obs}-Q_{sim}}{Q_{obs}}\right|$$

Where n is the number of observed data, Q_obs_ and Q_sim_ are the observed and simulated streamflow data respectively.

### 2.6 Future hydrological drought characterization

There are several hydrological drought indicators applied in many countries such as Palm Hydrological Drought Severity (PHDS), Surface Water Supply Index (SWSI), and Streamflow Drought Index (SDI). However, the selection of these indicators depends on their input data requirement, simplicity, widely practiced, etc. For example, PHDS and SWSI require at least three input data and for future drought analysis, all those required data could not be easily predicted. Accordingly, the streamflow drought index (SDI) is selected for this study to characterize future hydrological drought trends in Ethiopia using forecasted streamflow data as an input. The analysis of SDI is given by Equation (5) below and Table 2 shows the hydrological drought severity levels using SDI.

5 $$SDI=\frac{Q-Q_{m}}{\sigma }$$

Where Q and Q_m_ are seasonal observed and mean streamflow, respectively and σ is the standard deviation of the observed streamflow.

**Table 2:** SDI values for different drought severity levels.

Drought Condition	Wet	Normal	Mild Drought	Moderate drought	Severe drought	Extreme drought
Value	≥ 1.5	−0.4–1.4	−0.5–−0.99	−1–−1.4	−1.5–−1.99	≤ −2

## 3 Results

### 3.1 Climate data bias correction

Historical and future climate data was downscaled from GCM and RCM. Finally, the data was extracted and bias-corrected using five techniques with the help of Climate Model data for the hydrologic modeling (CMhyd) tool. It is found that Linear Scaling (LS) and power transformation (PT) have a minimum error. From the three selected models, the result shows that RACMO regional climate model (RCM) has good performance using the linear scaling bias correction technique. Except for the Omo Gibe River basin, all river basins satisfied that the RACMO model has a minimal error using the linear scaling bias correction technique whereas the Omo Gibe River basin has a minimum error using the RCA4 model, and the Linear Scaling bias correction technique also had a good performance. From Table 3, it is observed that power transformation has minimum error compared to linear scaling for MIROC 5. But for RACMO and RCA4, linear scaling has a minimum error of bias. Therefore, the linear scaling technique has good performance for bias correction of downscaled climate data by reducing the error below 2% and producing better climate-simulated outcomes in all river basins as shown in Table 3.

**Table 3:** Comparing a bias correction technique and climate-generating models.

Basin	Bias correction Technique	Model Error (%)		
		MIROC5	RACMO	RCA4
Abbay	Linear Scaling	2.43	1.67	1.89
	Power Transformation	2.31	1.89	2.17
Awash	Linear Scaling	3.57	1.44	1.96
	Power Transformation	3.06	1.48	1.90
Baro	Linear Scaling	2.14	1.12	1.22
	Power Transformation	1.95	1.28	1.29
Genale Dawa	Linear Scaling	4.00	1.54	2.63
	Power Transformation	3.93	1.68	2.53
Omo Gibe	Linear Scaling	2.55	2.43	2.01
	Power Transformation	2.50	2.37	2.03
	Linear Scaling	3.41	1.26	1.91
Rift Valley	Power Transformation	3.31	1.27	1.68
Tekeze	Linear Scaling	1.67	1.30	1.57
	Power Transformation	1.47	1.60	1.70
Wabishebele	Linear Scaling	5.88	2.37	3.98
	Power Transformation	7.06	2.80	3.43

### 3.2 Artificial neural network architecture development and streamflow forecasting

Recently R and Python software are used in many water resources studies such as flood inundation analysis, sediment prediction, low flow, and high flow forecasting, water quality analysis, natural disasters monitoring, and mitigation measure development. Studies indicated that python software becomes more efficient than R and is applicable in many data-driven environments [68]. Therefore, in this study python software was applied to forecast future streamflow using generated monthly precipitation from Regional Climate Model (RCM) as input for Back Propagation Multi-Linear Perceptron (BPMLP). Therefore, input – the hidden – output layer architecture relation was developed by trial-and-error approach until the model performed to the acceptable range.

The number of trials depends on the number of epochs, batch size per iteration, and the number of perceptions in a hidden layer. The epochs and batch size were tested from 50, 100, 1000 and 16, 32, 64, 128, respectively. From many trials, 100 epochs and 64 batches size gives a good performance. Then by fixing those values for all stations, the trail was repeated by changing the number of perceptron in the hidden layers. Table 4 shows the number of trials that found a good performance in line with selected epoch, batch size, and perceptron for different stations in Ethiopia and their corresponding statistical value of observed and simulated streamflow, respectively.

**Table 4:** Parameter values used to train and test hydrometeorological data sets in ANN.

Stations	Number of trials	Epochs	Batch size	Hidden layer Perceptron	Q_obs_ mean	σ_obs_	Q_sim_ mean	σ_sim_
Gummera	5	100	64	8	34.5	9.9	42.3	8.3
Gilge Belles	11	100	64	7	17.8	4.5	16.2	1.8
Kessie	13	100	64	8	523.7	166.5	698.9	102.6
Border	13	100	64	6	1675.7	325.5	2809.3	328.6
Awash7	4	100	64	6	57.6	17.5	65.4	6.8
Hombel	6	100	64	16	41.7	10.6	45.7	10.9
Gambela	7	100	64	8	398.5	94.3	393.2	31.6
Sorie	9	100	64	16	50.8	12.6	49.3	5.1
Assendabo	8	100	64	12	1217.5	309	1501.8	243.1
Gojeb	7	100	64	4	1845.9	433.9	1470.2	254.9
Embamadre	3	100	64	10	258	104.2	431.2	23.94
Wabishebele	5	100	64	9	7.4	2.1	9.1	1.1
Leliso	6	100	64	7	3.8	1.3	3.1	0.33

Table 5 clearly shows that relatively humid climate basins such as Abbay, Awash, Baro, Omo Gibe, and Tekeze have good prediction performance whereas the arid basins like Genale Dawa, Wabishebele and Rift valley has low prediction performance. The reason is that the hydroclimatic variables such as precipitation and streamflow variability is high in arid areas than in humid areas. The correlation between annual precipitation and mean annual streamflow for humid and arid basins of this study was found 0.55 to 0.77 and 0.23 to 0.52, respectively. As a result, forecasting future streamflow using highly varied precipitation data directly affects hydrological drought prediction and water resource management. This result agreed with previous drought predictions in using forecasted streamflow in Iran by Dasstorami. M.T and Afkhami. H (2011) [36]. From Table 5, it is understood that Abbay, Baro, and Omo Gibe River basins resulted in excellent streamflow forecasting performance compared to the remaining river basins. As shown in Table 1, those basins received annual precipitation above 1000 mm. Even though the RMSE and MAPE values for three arid river basins (Genale Dawa, Rift Valley, and Wabishebele) are acceptable, the R^2^ value is minimum. This indicates that ANN is suitable for humid and temperate climate zones to forecast streamflow from precipitation data.

**Table 5:** Statistical comparison of ANN forecasting performance.

River basin	Station	Modell Architecture	RMSE	MAPE	R^2^
Abbay	Gummera	3, 8, 1	1.9	1.1	0.9
	Gilgel Belles	3, 7, 1	0.83	0.47	0.83
	Kessie	3, 8, 1	3.6	2.3	0.81
	Border	4, 6, 1	11	8.32	0.84
Awash	Awash7	4, 6, 1	3.1	2.1	0.68
	Hombel	3, 16, 1	2.3	1.4	0.87
Baro	Gambela	2, 8, 1	16.1	11.6	0.77
	Sorie	3, 16, 1	2.03	1.4	0.83
Genale Dawa	Halewe	3, 8, 1	4.98	3.26	**0.59**
	Weib	2, 4, 1	12.45	9.4	**0.2**
Omo Gibe	Assendabo	3, 12, 1	6.5	4.7	0.77
	Gojeb	2, 4, 1	7.2	4.7	0.75
Rift Valley	Wosha	3, 8, 1	0.14	0.13	**0.24**
	Dedessa	3, 8, 1	1.31	0.89	**0.48**
Tekeze	Embamadre	4, 10, 1	12.5	8.8	0.7
Wabishebele	Wabishebele	2, 9, 1	5.66	4.22	**0.54**
	Leliso	3, 7, 1	1.82	1.29	**0.55**

Where 3, 8, and 1 indicate the number of input variables, Hidden perceptron, and output variable, respectively.

**Fig. 3:**
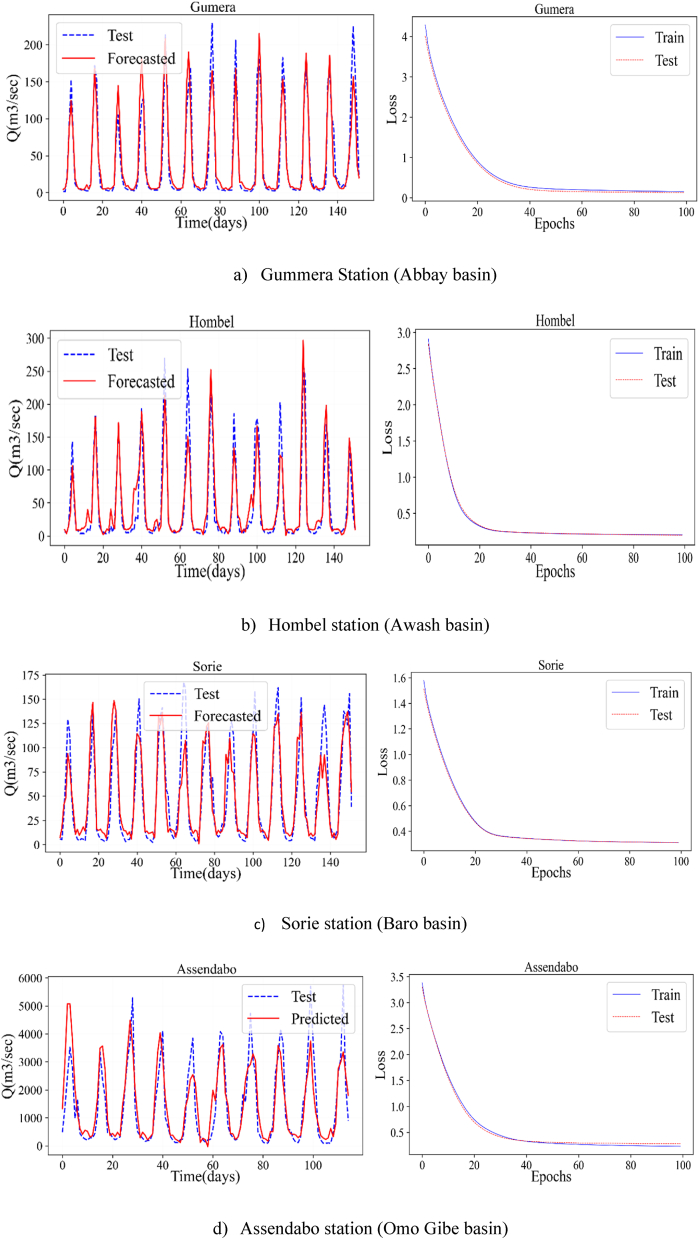
Observed and forecasted streamflow time series plot using ANN in Python software.

Fig. 3 (a, b, c, d) shows the observed versus forecasted streamflow time series graph for Abbay, Awash, Baro, and Omo Gibe River basins, respectively, developed using python software and the ANN model. The result revealed that the observed and simulated fitted with good performance. The analysis was computed using precipitation data as input and streamflow data as output in the ANN model setup. From this analysis, it is observed that streamflow forecasting from precipitation data has significant relation. As shown in Fig. 3, the loss decreases as the epoch increases in all selected streamflow stations. For a selected 100 epoch and 64 batch sizes, the train and test were best performed. The ultimate goal of forecasting streamflow in this study is to see the future hydrological drought trend in Ethiopia.

### 3.3 Future hydrological drought characterization

**Fig. 4:**
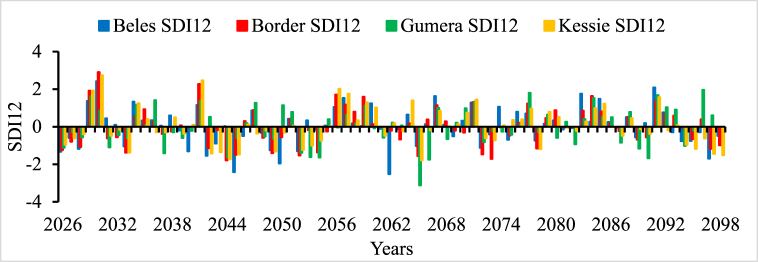
Future hydrological drought time series in Abbay river basin.

**Fig. 5:**
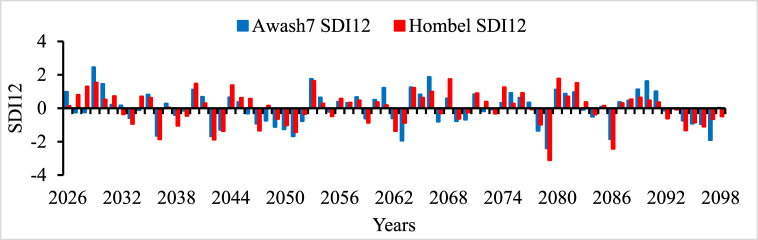
Future hydrological drought time series in Awash river basin.

**Fig. 6:**
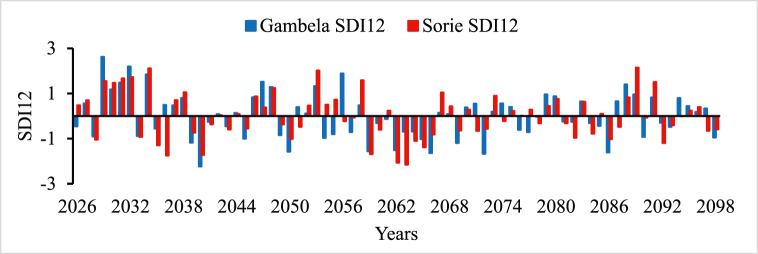
Future hydrological drought time series in Baro Akobo river basin.

**Fig. 7:**
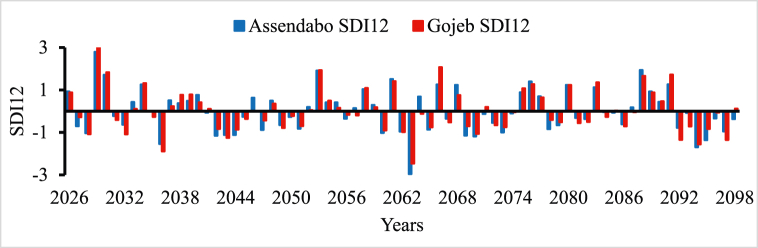
Future hydrological drought time series in Omo Gibe river basin.

**Fig. 8:**
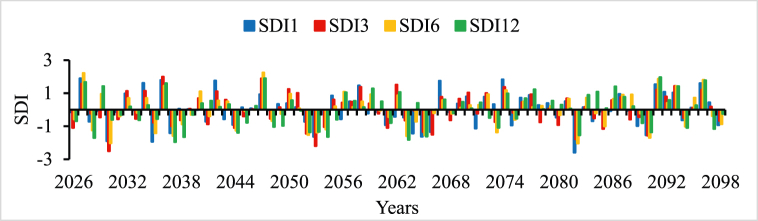
Future hydrological drought time series in Tekeze river basin.

**Fig. 9:**
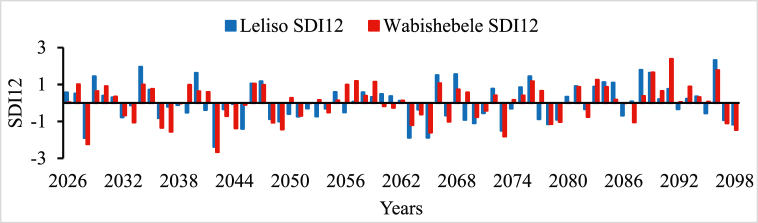
Future hydrological drought time series in Wabishebele river basin.

In all river basins except Genale Dawa and Rift Valley basins, the performance of streamflow forecasting using ANN indicated the possibility of using simulated streamflow time series to predict future hydrological drought conditions. Therefore, the forecasted streamflow was directly used as input for the DrinC model to predict future hydrological drought analysis. But in this study, Genale Dawa and Rift Valley basins were excluded due to the low performance of streamflow forecasting results. Therefore, the future hydrological drought analysis was conducted for six river basins as shown in Fig. 4, Fig. 5, Fig. 6, Fig. 7, Fig. 8, Fig. 9. The analysis was considered for four-time scales; monthly (SDI1), seasonal (SDI3), half-year (SDI6), and annual (SDI12) time scales. The result revealed that the frequency of drought occurrence is high for monthly (SDI1) and seasonal (SDI3) time scales compared to half-year and annual time scales (see Fig. 8). However, the duration of severe and extreme drought is high for annual time-scale analysis. Therefore, for this study, the result and discussion part focused on the annual (SDI12) time scale. Future hydrological drought condition in Ethiopia is analyzed for each river basin separately.

In the Abbay river basin, four streamflow was considered for future drought analysis and the result indicated that the probability of severe and extreme drought occurrence from 2026 to 241 was less in all stations. But in 2026, 2031, 2033, 2037, and 2040 moderate drought events are expected in some stations in the basin. However, Fig. 4 shows that 2042 to 2045, 2049/2050, 2052 to 2054, 2062, 2065, 2072/2073, and 2097/2098 are the most expected moderate to extreme drought years in the Abbay river basin. The most severe magnitude and frequency were identified at Gummera and Beles streamflow stations. The analysis indicated that the probability of severe drought occurrences in the future is more in the Abbay river basin compared to other river basins.

The Awash river basin is dominantly utilized for irrigation, hydropower, water supply, and other services compared to other river basins in Ethiopia. Therefore, in the future due to unlimited population increment, water sharing has become a significant issue in this basin. So, to manage this challenge, future drought analysis and investigation in the Awash river basin plays a great role. Fig. 5 indicated that 2036, 2042, 2063, 2079, 2086, and 2097 are severe and extreme drought events in the future in the Awash River basin. 2042, 2063, and 2097 are the common severe drought events for both Abbay and Awash river basins. This implies that a strong water resource management strategy, early drought warning system development, and good drought preparedness policies are important in those basins in the future. The awash river basin is a landlocked river and is more utilized for multipurpose. Water sharing and water stress are common problems in this river basin. In the future, climate change and drought will aggravate the water-sharing problem. Therefore, a strong water resource management system and systematic water allocation approaches are needed.

Baro Akobo and Omo Gibe River basins are received rainfall twice a year and have good climate condition compared to other river basins [69,70]. Although they received good rainfall, hydroclimatic disasters such as floods and droughts occurred in different parts of the basin [69]. But comprehensive drought studies were yet studied in both Baro Akobo and Omo Gibe River basins. Fig. 6, Fig. 7, respectively indicated that the frequency of severe and extreme drought occurrence is more in the Baro river basin than in the Omo Gibe River basin. The future drought analysis implies that 2036, 2050, 2059, 2066, 2072, and 2086 are severe drought years and 2040, 2062, and 2063 are extreme drought years for the Baro river basin whereas 2036 and 2094 are severe and 2063 are extreme drought years for Omo Gibe basin, respectively. Baro Akobo river is yet utilized but in the Omo Gibe basin there are few hydropower plants and irrigation projects are implemented in different tributaries of the main rivers; Aseendabo and Gojeb. The drastic drought in 2018 in the southern parts of Ethiopia affected the Gibe I and II hydropower plant production. During this year, the two hydropower plants produced energy below their expected potential. In the future, some hydropower plants are planned in the Baro Akobo river basin and it needs attention to consider the impact of drought resulting from climate change. Unless the construction and implementation of big developmental projects without detailed information the future hydroclimatic conditions will result in national economic crises.

In the Tekeze river basin, a single station data at Embamadre station was used to estimate SDI values at different time scales as shown in Fig. 8. Historically northeast part of Ethiopia is frequently affected by prolonged drought phenomena [71,72]. Gemeda (2022) also found that the northeast and southeast are the most drought-prone areas in Ethiopia [73]. Since the Tekeze river basin is located in the northern parts of the country and the result of this study also revealed the previous studies. The annual time scale (SDI12) results, 2028, 2037, 2038, 2054, 2063, 2065, and 2082 are the expected severe drought events in the Tekeze river basin. Based on the seasonal analysis, 2030 and 2053 were the expected extreme drought years. However, in hydrological drought analysis long time scale, 12 months and above is important to formulate the drought preparedness strategies and mitigation measurement development. But the seasonal time scale result has good importance in meteorological drought analysis.

Wabishebele river basin is one of the arid climate zone basins in Ethiopia. Future hydrological drought analysis was explored in this basin at two stations as shown in Fig. 9. It is found that 2036, 2037, 2063, 2065, and 2072 are severe drought years whereas 2028 and 2042 are the most extreme drought years in the future in Wabishebele Basin (see Fig. 9).

In this study, the future hydrological drought analysis was explored from 2026 to 2099 (74 years). Table 6, Table 7 show the expected future drought occurrence years at different streamflow stations in all river basins and a summary of each drought condition in a specific river basin in terms of percentage, respectively. The result also revealed that the frequency of moderate drought is increased in all river basins compared to severe and extreme drought occurrences. On the other hand, the occurrence of extreme drought is low compared to the last historical drought events in Ethiopia. For the case of the Tekeze river basin, a single streamflow station was used due to a lack of long-time recorded data as a result the probability of severe and extreme drought existence is also minimum (See Table 6).

**Table 6:** Summary of expected future drought years in Ethiopia at different river basins.

Basins	Stations	Moderate	Severe	Extreme
Abbay	Beles	2026, 2028, 2033, 2040, 2049, 2052, 2065, 2072, 2097	2042, 2050	2045, 2062
	Border	2026, 2028, 2033, 2042, 2045, 2054, 2072, 2078, 2097	2044, 2052, 2065, 2073	
	Gummera	2026, 2031, 2037,2052, 2089, 2094	2044, 2053,2054, 2066, 2090	2065
	Kessie	2026, 2033, 2042, 2043, 2045, 2049, 2052, 2053, 2065, 2078, 2096, 2097	2044, 2098	
Awash	Awash7	2043, 2049, 2052, 2078	2036, 2042, 2051, 2063, 2086, 2097	2079
	Hombel	2038, 2043, 2047, 2050, 2051, 2062, 2094, 2096	2036, 2042	2079, 2086
Baro Akobo	Gambela	2039, 2045, 2065, 2069	2050, 2059, 2062, 2066, 2072, 2086	2040
	Sorie	2028, 2035, 2050, 2064, 2065, 2086, 2092	2036, 2040, 2059	2062, 2063
Omo Gibe	Assendabo	2028, 2042, 2043, 2044, 2060, 2069, 2070, 2073, 2095	2036, 2094	2063
	Gojeb	2028, 2032, 2043, 2070, 2092, 2097	2036, 2094	2063
Tekeze	Embamadre	2044, 2048, 2052, 2053, 2073, 2090, 2094, 2097	2028, 2037, 2038, 2054, 2063, 2065, 2082	
Wabishebele	Leliso	2045, 2049, 2070, 2078, 2098	2028, 2063, 2065, 2073	2042
	Wabishebele	2033, 2036, 2044, 2063, 2067, 2078, 2079, 2087, 2097, 2098	2037, 2048, 2049, 2065, 2073	2028, 2042

**Table 7:** of drought occurrence per year based on the number of drought years.

Drought condition	Number of drought years in each River Basins					
	Abbay	Awash	Baro Akobo	Omo Gibe	Tekeze	Wabishebele
Moderate	17	11	10	13	8	13
Severe	11	6	8	2	7	7
Extreme	3	2	3	1	0	2
Percentage of occurrence per year (%)						
Moderate	22.97	14.86	13.51	17.57	10.81	17.57
Severe	14.86	8.1	10.81	2.7	9.46	9.46
Extreme	4.05	2.7	4.05	1.35	0	2.7

## 4 Discussions

Climate change aggravates hydroclimatic hazards such as floods and droughts [74]. To monitor and develop appropriate mitigation measures for those events, downscaling projected climate data plays a great role. Since Ethiopia is located under the Africa domain (AFR – 44) and the historical and future daily precipitation data were downscaled from three regional models (MIROC5, RACMO, and RCA4) using the RCP4.5 climate change scenario. Even though the regional climate data has minimal error compared to Global Climate Model (GCM), bias correction of raw data further reduced the projection error. In this study, five bias correction methods were applied and the result revealed that linear scaling (LS) has good performance and reduced the error below 2%. This result agreed with Nurul Nadrah's (2018) finding, he found that LS reduced climate-simulated error reduced below 3% [75].

Hydrological drought forecasting depends on the future variability of surface water availability. But the big challenge for hydrologists is forecasting streamflow appropriately with time and space. However, nowadays artificial intelligence and machine learning becomes more popular in water resources investigation to predict streamflow in the future using data-driven models. Therefore, ANN was used in this study to forecast the streamflow of eight river basins in Ethiopia for 17 streamflow stations. The result shows that about 13 streamflow stations from Abbay, Baro Akobo, Omo Gibe, Awash, and Tekeze river basins have given good performance with an average R^2^ value of 0.85, 0.8, 0.76, 0.75, and 0.7, respectively. But for the most semi-arid and arid river basins such as Genale Dawa, Rift Valley, and Wabishebele river basins the simulated performance result of the ANN model was low. Therefore, in this study the analysis of future hydrological drought in Ethiopia was focused on six selected river basins; Abbay, Awash, Baro Akobo, Omo Gibe, Tekeze, and Wabishebele. The result revealed that Ethiopia will be stricken with severe and extreme droughts in the coming future. The most common extreme drought years identified using SDI at a 12-month time scale in the future are 2036, 2042, 2044, 2062, and 2063. Most previous drought forecasting studies were assessed for a short time scale, 1-month, 2-month, or 3-month ahead [[76], [77], [78]]. But this study assessed long-time drought forecasting using downscale climate data from the RCP4.5 climate change scenario and by forecasted streamflow using ANN in python software. This result agreed with the approach of [79] predicting future drought in Korea using the RCP8.5 climate change scenario.

Now a day the government of Ethiopia is on the way to implementing Green Legacy to reduce the impact of climate change and the corresponding drought. But still, water resource management and hydrological event analysis need further investigations to alleviate poverty and the consequence of worth drought impact on society. Therefore, this study is a starting point for future further studies, policymakers, water resource managers, decision-makers, and drought mitigation measurement development strategies. Recently using Artificial Intelligence models and machine learning becomes crutial to develop drought early warning system. So, policy makers, planners, river basin developers of the nation have to be give emphasis to those data driven models in climate change and hydrological analysis studies.

## 5 Conclusion

The impact of climate change on streamflow and its consequences can be analyzed using projected climate data from GCM and RCM with observed hydroclimatic data. To minimize the effect of hydrological events on water resource projects, drought forecasting and monitoring system development have importance for policymakers, drought preparedness, and water resource management sectors. This study was standing intending to forecast long-term hydrological drought in Ethiopia using observed and projected climate data. A data-driven model such as ANN improves the problems related to non-linear and stationarity cases in water resource management analysis. The result of this study also indicated the possibility of forecasting long-time streamflow time series using precipitation data as an input for the ANN model. It is observed that humid and temperate climate zones can result in good streamflow forecasting performance compared to semi-arid and arid areas. In Ethiopia, Abbay, Baro Akobo, and Omo Gibe River basins received mean annual precipitation of above 1200 mm, and the Awash and Tekeze river basins received mean annual precipitation between 700 and 1100 mm. Streamflow forecasting from those areas using the ANN model corresponding to areas having high precipitation results in acceptable R^2^ values. Genale Dawa, Rift Valley, and Wabishebele river basins are located in the lowland parts of Ethiopia and received low annual precipitation. The source of streamflow for these basins is Bale Mountain and other highland areas of the country. As a result, the correlation between precipitation and streamflow data is very low. Therefore, the result revealed that forecasting streamflow directly using precipitation data will not give good performance in arid areas. The future hydrological drought analysis result indicates that there will be frequent moderate drought events in all river basins and the 2030s, 2040s, and 2060s were identified as the most expected severe and extreme drought event occurrence years. High population growth rates and dynamic climate change will increase water stress and water-sharing conflict in resource-limited areas in the future. In addition to this, drought has a worthwhile impact on the overall economic growth of the nation. Therefore, the government and policymakers should have to plan long-term drought preparedness and mitigation measures to minimize the risk associated with expected hideous drought events.

The government of Ethiopia is practically focused on drought crisis management than risk management. Drought crisis management is a short-term drought preparedness mechanism by supplying food and water to drought-prone areas. This kind of drought preparedness is not bringing a sustainable solution. Therefore, in the future the government should shift to a new paradigm by developing a national drought policy. Currently, a proactive approach is recommended as a short-term and long-term drought mitigation mechanism. A 2021 and 2022 Green Legacy in Ethiopia is a good initiative to prevent the climate change impact on water resources such as rivers, lakes, and reservoirs as a long – term mitigation approach. So, Afforestation, and the development of alternative energy sources from solar and wind have to be strongly encouraged in the future.

## Declarations

### Author contribution statement

Kassa Abera Tareke, Msc: Conceived and designed the experiments; Performed the experiments; Analyzed and interpreted the data; Contributed reagents, materials, analysis tools or data; Wrote the paper.

### Funding statement

This research did not receive any specific grant from funding agencies in the public, commercial, or not-for-profit sectors.

### Data availability statement

Data will be made available on request.

### Declaration of interest's statement

The authors declare no competing interests.
